# Pharmacists practising in family medicine groups: An evaluation 2 years after experiencing a virtual community of practice

**DOI:** 10.1177/17151635211049235

**Published:** 2021-10-15

**Authors:** Line Guénette, Anne Maheu, Marie-Claude Vanier, Nicolas Dugré, Léonie Rouleau, Jacynthe Roy-Petit, Lyne Lalonde

**Affiliations:** Faculty of Pharmacy, Laval University; CHU de Québec Research Centre, Québec City; Centre intégré universitaire de santé et de services sociaux de la Capitale-Nationale, Québec City; Centre intégré universitaire de santé et de services sociaux du Nord-de-l’île-de-Montréal; Faculté de pharmacie, Université de Montréal, Montréal; Faculté de pharmacie, Université de Montréal, Montréal; Centre intégré de santé et de services sociaux de Laval, Laval; Centre intégré universitaire de santé et de services sociaux du Nord-de-l’île-de-Montréal; Faculté de pharmacie, Université de Montréal, Montréal; Faculté de pharmacie, Université de Montréal, Montréal; Faculté de pharmacie, Université de Montréal, Montréal; Centre intégré de santé et de services sociaux de l’Estrie-CHUS, Sherbrooke; Québec; Faculté de pharmacie, Université de Montréal, Montréal

## Abstract

**Background:**

In 2018, a virtual community of practice (CoP) for pharmacists working in family medicine groups (FMGs) in Quebec province was developed. The aim of this CoP—called *Réseau Québécois des Pharmaciens GMF* (RQP GMF)—was to foster best practices by supporting FMG pharmacists. This study assesses the processes and outcomes of this CoP 2 years after its creation.

**Methods:**

We performed a cross-sectional web-based study from March to May 2020. All FMG pharmacists who were registered as members of the RQP GMF (*n* = 326) were sent an invitation via a newsletter. The link to the questionnaire was also publicized in the CoP Facebook group. The questionnaire comprised a 38-item validated instrument assessing 8 dimensions of the CoP. A descriptive analysis was performed.

**Results:**

A total of 112 FMG pharmacists (34.4%) completed the questionnaire. Respondents agreed that the RQP GMF was a joint enterprise (mean score, 4.18/5), that members shared their knowledge (mean score, 3.94/5) and engaged mutually (mean score, 3.50/5) and that the RQP GMF provided support (mean score, 3.92/5) and capacity building (mean score, 4.01/5). In general, they were satisfied with the implementation process (mean score, 3.68/5) and with activities proposed (mean score, 3.79/5). A lower proportion of respondents agreed that their participation in the RQP GMF generated external impacts, which led to a smaller mean score (3.37/5) for this dimension.

**Conclusion:**

The RQP GMF, one of the first communities of practice for pharmacists practising in family medicine groups, attained most of the objectives initially intended by the CoP. These results will facilitate the adaptation of processes and activities to better fulfil members’ needs. *Can Pharm J (Ott)* 2021;154:xx-xx.

**Knowledge Into Practice**

- Health care professionals with common interests are increasingly using online media to create virtual communities with the objective of building and sharing knowledge and consequently improving practices.
- The community of practice of family medicine group (FMG) pharmacists attained most of its objectives 2 years after its creation, as respondents thought it was a joint enterprise, that members shared their knowledge and that the resources provided enabled support and were capacity building.
- Although evidence suggests that clinicians prefer to use virtual CoPs to communicate within a clinical specialty, diversity, especially with community pharmacists, could stimulate intraprofessional collaboration and learning from both types of pharmacy practice.
- Participating in the community of FMG pharmacists was largely viewed as beneficial for members and their organization and most respondents would recommend it to other FMG pharmacists.
- Face-to-face or online meetings or training could be offered to build a sense of belonging, relationships and trust, so that FMG pharmacists fully engage in the community of practice.

**Mise En Pratique Des Connaissances**

- Les professionnels de la santé qui ont des interest communs utilisent de plus en plus les médias en ligne afin de créer des communautés virtuelles ayant pour objectif d’acquérir et de partager des connaissances, et par conséquent d’améliorer les pratiques.
- La communauté de pratique des pharmaciens du groupe de médecine de famille (GMF) a réalisé la plupart de ses objectifs 2 ans après sa création, car les répondants ont estimé qu’il s’agissait d’une entreprise commune, que les membres partageaient leurs connaissances et que les ressources fournies permettaient de soutenir et de renforcer leurs capacités.
- Bien que les preuves suggèrent que les cliniciens préfèrent utiliser une communauté de pratique virtuelle pour communiquer au sein d’une spécialité clinique, la diversité, notamment pour les pharmaciens communautaires, pourrait favoriser la communication intraprofessionnelle et l’apprentissage pour les deux types de pratique pharmaceutique.
- La participation à la communauté de pharmaciens du GMF a été largement considérée comme bénéfique pour les membres et leur organisation, et la plupart des répondants la recommanderaient à d’autres pharmaciens du GMF.
- Des réunions ou des formations en personne ou en ligne pourraient être proposées pour créer un sentiment d’appartenance, de confiance et des relations afin que les pharmaciens du GMF s’engagent pleinement dans la communauté de pratique.

## Introduction

In Canada and elsewhere, nondispensing pharmacists practising in multidisciplinary primary health care teams, such as family medicine groups (FMGs), are becoming more common.^1-3^ In the province of Quebec, these pharmacists are usually co-located in the clinic, and they are expected to work with other health care professionals in the provision of direct patient care. Half of these FMGs hire only one pharmacist on their team,^3^ and funding provided to hire pharmacists depends on the size (number of patients registered to the clinic) of the FMG. It is well documented that this new type of practice can be challenging for pharmacists,^4,5^ especially if this is a new experience for them and if they are the only pharmacist on the team. Joining a practice support network is strongly recommended to ease integration into primary care teams.^6^

Virtual communities of practice (CoPs) could provide this support to FMG pharmacists across the province. CoPs are learning communities focusing on a domain of common interest, with the objective of building and sharing knowledge and consequently improving practice.^7,8^ Health care professionals, including pharmacists, are increasingly using CoPs and online media to create virtual communities.^8-11^ Our research group has developed a virtual CoP for FMG pharmacists called the *Réseau Québécois des Pharmaciens GMF* (RQP GMF).^12^ As suggested by several authors,^7^ we first performed a needs assessment study describing FMG pharmacists’ characteristics, practices and settings and providing insights into their challenges to develop a CoP adapted to their needs.^3^ We also formulated a clear purpose, which was to support integration of pharmacists in FMGs, enable networking among FMG pharmacists and facilitate knowledge sharing and best practices (at both the clinical and organizational levels).^12^ The virtual CoP is hosted on an existing practice-based research network—the STAT network (www.reseaustat.ca). Activities and tools offered are described in detail in another publication.^12^ In brief, newsletters are regularly sent to members as a way to inform and engage them in the CoP. Tools developed by members are sought and shared on the website to support clinical practice and organization in the FMG. Finally, a directory of FMG pharmacists has been developed and is regularly updated. The publication of this directory on the RQP GMF website was seen as a first step to connect community pharmacists with their FMG colleagues and to foster intraprofessional collaboration. The aim of the present study was to formally assess the processes and outcomes of this CoP at 2 years after beginning its activities. We also aimed to describe the characteristics of pharmacists practising in family medicine groups, their practice and settings.

## Methods

### Study design and population

We performed a cross-sectional web-based study among FMG pharmacists who were registered as members of the RQP GMF. Registration was defined as having given contact information in the RQP GMF registry or receiving the RQP GMF newsletters. In March 2020, all 326 members were sent an invitation via a specific newsletter, and the link to the questionnaire was also publicized in the RQP GMF Facebook group. Seven reminders were sent via newsletters and Facebook publications. We also asked our working group of FMG pharmacists to assist with the recruitment, as the invitation was sent just 2 days before lockdown by local authorities due to the COVID-19 pandemic. Respondents completing the questionnaire were deemed to have given consent. The study was approved by the *Comité d’éthique de la recherche en santé* (CERES) of Montreal University (#18-041-CERES-D) and by the Ethics Committee of Laval University (#2018-079).

### Data collection and variables

The questionnaire was available from March 10 to May 19, 2020. It was developed and validated in collaboration with the Method Development platform of the Quebec-SPOR SUPPORT Unit. Questions were grouped into 3 sections: 1) information about pharmacists and their FMG (*n* = 15; e.g., age, sex, number of years in practice, type of practice outside the FMG, FMG type, number of pharmacists in the FMG, hours with a pharmacist in the FMG); 2) satisfaction regarding their activities in the FMG and the level of collaboration (*n* = 7; assessed with Likert scales); and 3) assessment of the CoP (*n* = 38). This last section was based on a literature review, insights from independent experts and debriefings with CoP members.^13^ The 38 items of this section were assessed with a 5-point Likert scale varying from totally disagree (score = 1) to totally agree (score = 5), as well as “nonapplicable,” and were categorized into CoP dimensions and 2 groups: generic items (pertinent to any CoP in any context) and specific items (pertinent to the RQP GMF). Dimensions evaluated were joint enterprise (*n* = 3), mutual engagement (*n* = 4), knowledge sharing (*n* = 2), social support (*n* = 3), capacity building (*n* = 5), implementation and evaluation (*n* = 2), facilitation/activities (*n* = 7) and external impact (*n* = 10). There were 2 additional items in an “other” category. Eleven FMG pharmacists validated each item’s relevance and clarity before its use in the present study.

### Analysis

We performed a descriptive analysis. For CoP dimensions, we also calculated a mean score for each item along with standard deviation (SD) and the mean of the mean scores for each dimension. Nonapplicable answers were removed from the denominator when scores were grouped.

## Results

### Characteristics of pharmacists and their FMG

Among the 326 FMG pharmacists who were registered as members of the RQP GMF in May 2020, 112 (34.4%) completed the online questionnaire. Most were women (75.9%), were younger than 40 years (58.9%), had a bachelor’s degree (49.1%) or a professional doctorate in pharmacy (PharmD: 30.4%) as their highest level of education, and practised as salaried community pharmacists (72.3%). A quarter of them (24.1%) had been practising pharmacy for fewer than 5 years. Respondents were practising in an FMG for a mean ± SD of 13.7 ± 8.0 hours a week. Most of them had 3 years or more of experience in an FMG (58.0%) and their affiliation with the FMG was self-employment (78.6%). Most of the settings were not university affiliated (83.0%), were located on 1 site (59.8%) and had had a pharmacist on the team for 2 to 5 years (67.9%). Half of them (49.1%) hired only 1 pharmacist. The mean number of hours per week for which a pharmacist was present in the FMG was 19.1 ± 9.9. The respondents and their FMG characteristics are presented in Table 1.

**Table 1:** Characteristics of pharmacists working in a family medicine group and their practice (total *n* = 112)*

FMG pharmacist characteristics	*n*	%
Sex		
Male	27	24.1
Female	85	75.9
Age		
<30 years	27	24.1
30-39 years	39	34.8
40-49 years	30	26.8
≥50 years	16	14.3
Highest pharmacy degree completed		
Bachelor	55	49.1
DESS	4	3.6
PharmD (entry level)	34	30.4
Master	17	15.2
Other	2	1.8
Time since obtaining practice licence in Quebec province		
<5 years	27	24.1
5-9 years	23	20.5
10-14 years	21	18.8
15-19 years	7	6.3
20-24 years	17	15.2
>24 years	16	14.3
Unknown	1	0.9
Experience in FMG		
<6 months	5	4.5
6-12 months	8	7.1
>1-2 years	34	30.4
3-5 years	56	50.0
>5 years	9	8.0
Experience in community pharmacy		
<6 months	11	9.8
6-12 months	2	1.8
>1-2 years	6	5.4
3-5 years	21	18.8
>5 years	72	64.3
Hours of presence in the FMG per pharmacist per week, mean ± SD	13.7 ± 8.0	
Other work setting^†^		
Community pharmacy (salary)	81	72.3
Community pharmacy (owner)	8	7.1
Health institution	17	15.2
FMG only	2	1.8
Other	10	8.9
Type of affiliation with the FMG		
Self-employed	88	78.6
Through a health institution	18	16.1
Through a community pharmacy	0	0.0
Other	6	5.4
Type of FMG		
FMG on 1 site	53	47.3
FMG multiple sites	31	27.7
FMG-U on 1 site	14	12.5
FMG-U multiple sites	5	4.5
FMG-R	9	8.0
Time with a pharmacist on the team		
<6 months	0	0.0
6-12 months	3	2.7
>1-2 years	14	12.5
>2-5 years	76	67.9
>5 years	19	17.0
Number of FMG pharmacists in the FMG		
1	55	49.1
2	38	33.9
≥3	19	17.0
Total number of hours per week a pharmacist is present in the FMG, mean ± SD	19.1 ± 9.9	
Estimated proportion of general practitioners referring patients to the pharmacist, mean ± SD	66.3 ± 23.3	

DESS, *Diplôme d’études supérieures specialisées* (specialized graduate studies); FMG, family medicine group; FMG-U, University-affiliated family medicine group; FMG-R, family medicine group - Réseau (Network) or super clinic; PharmD, professional doctorate in pharmacy.

* Values are expressed as *n* (%) unless otherwise noted.

† Six pharmacists chose more than 1 answer (*n* = 118).

### Satisfaction of pharmacists with their practice and professional collaboration in the FMG

**Figure 1:**
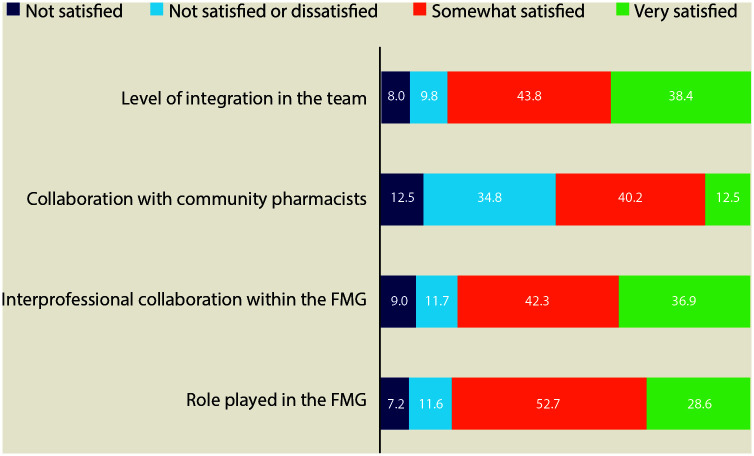
Satisfaction of pharmacists with their practice and professional collaboration in the family medicine group (FMG)

Pharmacists were satisfied with their integration, their role and the degree of interprofessional collaboration in the FMG (Figure 1). However, they were less satisfied with the degree of collaboration with community pharmacists. Twenty-three percent of respondents estimated that community pharmacists never communicated with them in a typical day. A significant proportion of them (68.8%) declared they were “very or extremely confident” with their capacity to play their role optimally in the FMG.

### Processes and outcomes of the CoP

Results of the processes and outcomes of the RQP GMF are presented in detail in Table 2. Respondents agreed that the RQP GMF was a joint enterprise (mean score, 4.18/5 ± 0.76), that members were sharing knowledge (mean score, 3.94/5 ± 0.81) and that the RQP GMF provided support (mean score, 3.92/5 ± 0.72) and capacity building (mean score, 4.01/5 ± 0.72). Respondents were less in agreement regarding whether there was mutual engagement among members of the RQP GMF (mean score, 3.50/5 ± 0.98). In general, they were satisfied with the implementation of the RQP GMF (mean score, 3.68/5 ± 0.78) and with activities proposed (mean score, 3.79/5 ± 0.98). A lower proportion of respondents agreed that there were some external impacts of their participation in the RQP GMF that led to a smaller mean score for this dimension (mean score, 3.37/5 ± 1.01). Finally, a large proportion of members surveyed agreed that participating in the RQP GMF was beneficial for them or their FMG (74.1%) and would recommend the RQP GMF to other FMG pharmacists (88.4%).

**Table 2:** Number and proportion of agreement to statements related to the processes and outcomes of the community of practice with mean score by item and by dimension (*n* = 112)

	Strongly agree or agree		Neutral or do not know		Disagree or strongly disagree		Nonapplicable		Mean score		
Processes and outcomes of the RQP GMF	*n*	%	*n*	%	*n*	%	*n*	%	Dimension	Item	SD
*Joint enterprise*									(4.18)		0.76
The RQP GMF takes into account the needs of its members	95	84.8	14	12.5	1	0.9	2	1.8		4.20	0.69
RQP GMF members help each other	83	74.1	24	21.4	4	3.6	1	0.9		3.98	0.80
I see the long-term value of the RQP GMF for its members	96	85.7	14	12.5	1	0.9	1	0.9		4.37	0.74
*Mutual engagement*									(3.50)		0.98
I am satisfied with my participation in the RQP GMF	53	47.3	34	30.4	15	13.4	10	8.9		3.46	0.92
I am motivated to participate in the RQP GMF	70	62.5	31	27.7	10	8.9	1	0.9		3.67	0.91
I am comfortable with sharing my knowledge, experiences and points of view with the members of the RQP GMF	68	60.7	21	18.8	21	18.8	2	1.8		3.56	1.01
I feel a sense of belonging to the RQP GMF	47	42.0	36	32.1	24	21.4	5	4.5		3.28	1.04
*Knowledge sharing*									(3.94)		0.81
The RQP GMF allows me to share my knowledge, experiences and points of view	67	59.8	29	25.9	9	8.0	7	6.3		3.70	0.87
The knowledge acquired at the RQP GMF can be used to develop new ways of doing things	92	82.1	15	13.4	1	0.9	4	3.6		4.17	0.69
*Social support*									(3.92)		0.72
My participation in the RQP GMF reduces my professional isolation	70	62.5	29	25.9	5	4.5	8	7.1		3.77	0.75
The RQP GMF atmosphere allows for openness and creativity	76	67.9	28	25.0	1	0.9	7	6.3		3.89	0.68
RQP GMF members make constructive comments (e.g., in the forum or on the Facebook group)	85	75.9	17	15.2	1	0.9	9	8.0		4.11	0.70
*Capacity building*									(4.01)		0.72
The RQP GMF allows me to learn about the experiences and views of other members	92	82.1	14	12.5	2	1.8	4	3.6		4.08	0.67
The RQP GMF helps me to compare ways of doing things (*n* = 110)	88	80.0	14	12.7	3	2.7	5	4.5		4.04	0.69
In the RQP GMF, sharing experiences and points of view confirms that my practice is adequate	79	70.5	25	22.3	1	0.9	7	6.3		3.85	0.60
The RQP GMF brings me new knowledge useful for my practice or for myself	92	82.1	10	8.9	6	5.4	4	3.6		4.11	0.79
My participation in the RQP GMF allows me to improve my skills or my practice	81	72.3	18	16.1	6	5.4	7	6.3		3.94	0.79
*Implementation and evaluation*									(3.68)		0.78
I am satisfied with the way RQP GMF members can connect with each other (*n* = 111)	77	69.4	21	18.9	9	8.1	4	3.6		3.79	0.84
There is a process to propose improvements of the RQP GMF	48	42.9	54	48.2	1	0.9	9	8.0		3.57	0.71
*Facilitation/activities*									(3.79)		0.98
I am satisfied with the activities offered by the RQP GMF	65	58.0	33	29.5	6	5.4	8	7.1		3.64	0.75
I am satisfied with the way RQP GMF members are invited to participate	67	59.8	30	26.8	10	8.9	5	4.5		3.67	0.83
I am satisfied with the frequency of the RQP GMF newsletters	91	81.3	13	11.6	3	2.7	5	4.5		4.10	0.75
I am satisfied with the content of the RQP GMF newsletters	85	75.9	14	12.5	7	6.3	6	5.4		4.08	0.86
I am satisfied with the platform (STAT Network) for hosting RQP GMF documents and tools	71	63.4	21	18.8	14	12.5	6	5.4		3.74	1.07
I am satisfied with the pharmacotherapeutic capsules (PharmAstuces)	76	67.9	22	19.6	1	0.9	13	11.6		4.15	0.80
I consult the directory of FMG pharmacists available on the STAT Network	42	37.5	15	13.4	37	33.0	18	16.1		3.10	1.32
*External impact*									(3.37)		1.01
My participation in the RQP GMF has increased my satisfaction at work or in doing other activities	45	40.2	42	37.5	13	11.6	12	10.7		3.38	0.83
My participation in the RQP GMF allows me to better integrate myself into my FMG	43	38.4	40	35.7	19	17.0	10	8.9		3.33	0.96
My participation in the RQP GMF allows me to carry out certain activities more effectively	58	51.8	32	28.6	12	10.7	10	8.9		3.56	0.87
I share the pharmacotherapeutic capsules with the members of my FMG	36	32.1	17	15.2	34	30.4	25	22.3		3.08	1.30
I use the tools developed by the RQP GMF to increase awareness of the role of the pharmacist in the FMG (*n* = 111)	44	39.6	21	18.9	28	25.2	18	16.2		3.22	1.15
The RQP GMF contributes to developing new practices within my FMG	46	41.2	33	29.5	20	17.9	13	11.6		3.29	0.99
My participation in the RQP GMF allows me to better define my service offer (*n* = 111)	58	52.3	29	26.1	13	11.7	11	9.9		3.55	0.95
Links between FMG pharmacists have been strengthened thanks to the RQP GMF	66	58.9	31	27.7	11	9.8	4	3.6		3.64	0.90
Links between FMG pharmacists and other pharmacists are facilitated thanks to the RQP GMF	37	33.0	45	40.2	26	23.2	4	3.6		3.15	0.99
Through being a member of the RQP GMF, I make useful new contacts for my FMG or for myself	53	47.3	32	28.6	16	14.3	11	9.8		3.45	0.98
*Other*									(4.12)		0.80
I really see the benefits of participating in the RQP GMF for my FMG or for myself	83	74.1	21	18.8	5	4.5	3	2.7		3.95	0.82
I would recommend the RQP GMF to other FMG pharmacists	99	88.4	7	6.3	3	2.7	3	2.7		4.28	0.75

FMG, family medicine group; RQP GMF, Réseau Québécois des Pharmaciens GMF.

## Discussion

Our objective was to assess the processes and outcomes of a virtual CoP of pharmacists working in an FMG at 2 years after its creation. We also aimed to describe the characteristics of pharmacists practising in family medicine groups, their practice and settings to appraise the evolution since the needs assessment carried out in 2018.^3^

We found that the RQP GMF attained most of the objectives intended by communities of practice.^7,8^ Respondents thought that the RQP GMF was a joint enterprise, that members shared their knowledge and that resources provided enabled support and were capacity building. An important dimension of CoP that could be further developed is mutual engagement among members. Among suggestions made by respondents at the end of the survey (results not presented), some expressed the need for face-to-face meetings. Prior studies also tend to support the importance of offline activities and face-to-face communication for building trustworthy relationships and establishing a sense of belonging among members of virtual communities.^7,14^

Although we performed a needs assessment as the first step in developing the RQP GMF,^3^ satisfaction with some of the activities proposed by the RQP GMF and perceived external impacts of participation could be improved. We observed that a fair proportion of respondents (around 10%) used the “nonapplicable” option for items related to participation (e.g., “I am satisfied with my participation in the RQP GMF,” “My participation in the RQP GMF has increased my satisfaction at work or in doing other activities”) or specific activities (e.g., “I am satisfied with the pharmacotherapeutic capsules,” “I consult the directory of FMG pharmacists available on the STAT Network”). This suggests that respondents did not participate or consult the tools and activities developed and shared through the RQP GMF. A low uptake by the target group and the fact that most contributions are attributed to a limited number of individuals have also been observed in other virtual communities^7,9^ including 1 primary care pharmacists community.^11^ Wenger et al.^15^ described 3 levels of participation in CoP: a core leadership group of active participants (10%-15%), a small active group who attend meetings regularly and participate in forums occasionally (15%-20%), and the rest of the members, who are peripheral and rarely participate.

The highest proportion of “nonapplicable” responses (22.3%) was observed for this item: “I share the pharmacotherapeutic capsules with the members of my FMG.” This might indicate that members are not comfortable sharing tools developed by the pharmacists’ CoP with other health care professionals. In their integrative review of virtual communities, Rolls et al.^9^ stated, “Current social networks in health care organizations are generally homophilous (i.e., individuals share common attributes) with strong professional boundaries.” They concluded that evidence suggests that clinicians prefer to use a virtual CoP to communicate within a clinical specialty, as most of those communities identified were for a specialty within a single discipline,^9^ although heterogeneity can be appreciated and foster learning.^16^ It is noteworthy that only 3 pharmacotherapeutic capsules had been published at the time of the survey. Moreover, some pharmacists indicated that the information communicated in the documents had already been discussed within their FMG. A qualitative study to further explore the results of the survey will be performed in the next months.

It is important to remember that one of the purposes of the RQP GMF was to support integration of pharmacists into FMGs. A mentorship program to assist pharmacists and to increase their confidence in their capacity to play their role optimally was offered. Tools to communicate pharmacists’ competencies to other team members and to promote the role of pharmacists in the FMG were developed and shared. These activities and tools were developed based on the needs expressed initially by FMG pharmacists^3^ and seem to have been successful, as pharmacists now report being satisfied with their integration, their role and the degree of interprofessional collaboration in the FMG. However, these needs may have shifted with increased experience. In the future, it will be important to prioritize unaddressed and new needs.

Among comments at the end of the survey, several respondents expressed the need for advanced clinical training, case discussions and discussions about pharmacotherapy. Trinacty et al.^11^ performed a qualitative content analysis of 1-year activities related to a listserv offered to members of the Canadian Primary Care Pharmacy Specialty Network. Those investigators found that discussions were often related to the care of patients with complex medical conditions and needs or as a forum for mentorship. The investigators also found that pharmacists practising primarily in family practice asked more questions than those from other areas of pharmacy practice.^11^ This emphasizes, as suggested in the literature, the need to encourage diversity among members. There should be varying demands and diverse expertise and levels of competency so that members can learn from others and share their expertise.^7^ Various participant roles have been suggested in the literature as being necessary for successfully managing virtual communities: 1) leaders (project manager, moderator/facilitator); 2) core members (subject experts, content coordinator); 3) support persons (mentors, those providing technical support); and 4) community members (active or nonactive, co-learners).^7^

As in 2018,^3^ community pharmacists rarely communicated with FMG pharmacists even if one of the first tools developed and shared by the RQP GMF was a directory of all FMG pharmacists with their contact information. We hypothesized that the directory was not publicized and known enough among community pharmacists, the target users. Tools could be developed to promote the use of this directory and so improve collaboration between pharmacists. This result also emphasizes the need to have common activities and places to exchange information, ideas or tools in order to build relationships and trust between community and FMG pharmacists.

Compared to 2018, a higher proportion of pharmacists had confidence in their capacity to play their role optimally. This could be the result of the CoP or it may simply be caused by respondents having more experience with this practice in 2020. Pottie et al.^17^ reported that pharmacists needed time to expand their knowledge and new skills to address family practice needs.

Our study was conducted with a thoroughly developed and validated questionnaire based on the conceptual framework proposed and revised by Wenger, the father of CoP.^8,13^ It assessed 8 dimensions that are crucial in communities of practice. The response rate (34.4%) was lower than first expected. However, considering that the survey was launched just 2 days before the first restrictions related to the SARS-COV-2 pandemic, we reached an appreciable proportion of all Quebec province FMG pharmacists. Apart from an expected greater experience in FMG, characteristics of the respondents were similar to those of 2018. We also had a smaller response rate from pharmacists affiliated with regional health authorities (*Centre intégré [universitaire] de santé et de services sociaux—CISSS* and *CIUSSS*). We hypothesize that these pharmacists were mobilized by their respective organizations and were less available from March to May 2020 due to the COVID-19 pandemic.

## Conclusion

This study assessed one of the first CoPs for pharmacists practising in family medicine groups with respect to the activities and tools developed in the first 2 years of its creation. Overall, the members were satisfied and participated in the community’s activities. The results will enable the adaptation of processes and activities to better fulfil members’ needs. Mutual engagement among members is a dimension that will have to be further developed. Other research is needed to determine whether the RQP GMF improves patient outcomes by facilitating professional support, knowledge transfer and evidence-based practice. ■
